# Technical note on the validation of a semi-automated image analysis software application for estrogen and progesterone receptor detection in breast cancer

**DOI:** 10.1186/1746-1596-6-6

**Published:** 2011-01-18

**Authors:** László Krecsák, Tamás Micsik, Gábor Kiszler, Tibor Krenács, Dániel Szabó, Viktor Jónás, Gergely Császár, László Czuni, Péter Gurzó, Levente Ficsor, Béla Molnár

**Affiliations:** 1 H-1063 Budapest, Podmaniczky u. 63, Hungary; 2 Ist Department of Pathology and Experimental Cancer Research, Semmelweis University, Budapest, Hungary; 3 3DHISTECH Ltd., Konkoly-Thege Miklós út 29-33, Building 18, Budapest H-1121, Hungary; 4 University of Pannonia, Faculty of Information Technology, I-012, Egyetem u. 10., 8200 Veszprém, Hungary; 5 Hungarian Academy of Sciences, Clinical Gastroenterology Research Unit, Budapest, Hungary

## Abstract

**Background:**

The immunohistochemical detection of estrogen (ER) and progesterone (PR) receptors in breast cancer is routinely used for prognostic and predictive testing. Whole slide digitalization supported by dedicated software tools allows quantization of the image objects (e.g. cell membrane, nuclei) and an unbiased analysis of immunostaining results. Validation studies of image analysis applications for the detection of ER and PR in breast cancer specimens provided strong concordance between the pathologist's manual assessment of slides and scoring performed using different software applications.

**Methods:**

The effectiveness of two connected semi-automated image analysis software (*NuclearQuant *v. 1.13 application for *Pannoramic*™ *Viewer *v. 1.14) for determination of ER and PR status in formalin-fixed paraffin embedded breast cancer specimens immunostained with the automated Leica Bond Max system was studied. First the detection algorithm was calibrated to the scores provided an independent assessors (pathologist), using selected areas from 38 small digital slides (created from 16 cases) containing a mean number of 195 cells. Each cell was manually marked and scored according to the Allred-system combining frequency and intensity scores. The performance of the calibrated algorithm was tested on 16 cases (14 invasive ductal carcinoma, 2 invasive lobular carcinoma) against the pathologist's manual scoring of digital slides.

**Results:**

The detection was calibrated to 87 percent object detection agreement and almost perfect Total Score agreement (Cohen's kappa 0.859, quadratic weighted kappa 0.986) from slight or moderate agreement at the start of the study, using the un-calibrated algorithm. The performance of the application was tested against the pathologist's manual scoring of digital slides on 53 regions of interest of 16 ER and PR slides covering all positivity ranges, and the quadratic weighted kappa provided almost perfect agreement (κ = 0.981) among the two scoring schemes.

**Conclusions:**

*NuclearQuant *v. 1.13 application for *Pannoramic*™ *Viewer *v. 1.14 software application proved to be a reliable image analysis tool for pathologists testing ER and PR status in breast cancer.

## Background

Early detection of breast cancer, one of the important causes of cancer morbidity worldwide [1], can significantly improve the survival probability of the patients [2,3]. Identifying molecules which regulate the growth of normal and transformed breast epithelium by means of immunohistochemistry, has been used as adjuncts for diagnostic, prognostic, and predictive decision-making [4]. The most established validated molecular markers predicting responsiveness of patients for molecular targeted therapy are estrogen (ER) and progesterone (PR) receptors [5] and the type-2 human epidermal growth factor receptor (HER2).

Large scale randomized clinical trials proved that patients with double positive (ER/PR) status are likely to benefit from endocrine/hormonal therapy. According to the recent ASCO-CAP guidelines, ER and PR status should be determined in all invasive breast cancers and recurrences [6]. However, the reliability of assay results depends on both the reproducibility of assay performance and its interpretation [7,8].

Standardized protocols used by automated immunostainers set up high standards in test reproducibility. Whole slide digitalization, supported by dedicated software tools allows image object quantization based on colour and intensity segmentation for unbiased analysis of immunostaining results [9].

Several validation studies of image analysis applications for the detection of ER and PR in breast cancer specimens have been published [10-16] and results provided strong concordance between the pathologist's manual assessment of slides and scoring performed by the different software applications.

Our survey was performed to test the effectiveness of two connected software application: *Pannoramic*™ *Viewer *v. 1.14 (hereinafter *PV*) and *NuclearQuant *application for *PV *v. 1.13 (hereinafter *NQ*) both manufactured by 3DHISTECH Ltd. (Budapest, Hungary). *PV *enables the visualization of digital slides, users being able to inspect and annotate (i.e. select certain regions of an image) digital slides. These editable (i.e. possibility for deletion, rename) annotations can be rectangular and/or free hand. *NQ *is an image-analysis software connected to *PV*, suitable for unbiased automated analysis of digital image objects based on colour, intensity and size. It detects and separates nuclei-shaped connected pixel sets (e.g. immunolabelled cell nuclei) on microscopic digital slides (*.mrxs file format) created using one of the digital slide scanners manufactured by 3DHISTECH Ltd. (i.e. *Pannoramic DESK*, *Pannoramic MIDI*, *Pannoramic *SCAN 150). For image standardization [17] Wallis image filters are used, to compensate rarely perceptible but possibly occurring local intensity deviations arising from unsuitable luminance and optical aberrations. *NQ *uses a "colour deconvolution" algorithm for the scoring process, which decomposes the RGB image into greyscale intensity images that represent the staining or dye concentration maps individually for each stain. Nuclei scoring is based on the intensity of connected pixel sets measured on the 3,3'-Diaminobenzidine (DAB) intensity image only. The nuclei detection is performed on the intensity normalized greyscale image, based on the morphometrical parameters, such as optimal roundness, average density and proper contrast of the intensity at the boundary. Scores are calculated for each detected object, based on the average intensity of the corresponding pixels of the intensity image. Users may filter object by size or shape, and can separate joint objects. The information necessary for the algorithm to be used is not saved on the image itself but are stored in Microscopic Image Segmentation Profile files (*.misp). The application can be run on whole slides or annotations, and the detected objects can be viewed, reclassified, relocated and visualized. Both research use applications are intended to support in vitro diagnostic decision-making, aiding pathologists in the detection, scoring, classification and counting of cells of interests.

The aims of our validation study were: 1) calibration of the algorithm for ER and PR detection built-in *NQ *on immunostained slides after whole-slide digitalization; and 2) assessment of equivalence of the semi-automated detection using the software application with the manual scoring of digital slides.

## Methods

### Samples, slide selection, digitalization and pre-quantization procedures

ER and PR stained IHC breast cancer slides from 16 cases (14 invasive ductal carcinoma Grade I-III and 2 invasive lobular carcinoma) were used from the archive of the 1st Department of Pathology and Experimental Cancer Research of the Semmelweis University, Budapest, Hungary. The formalin-fixed paraffin embedded specimens were stained (as defined in the manufacturer's staining protocol) on a Bond-max™ fully automated staining system (Leica Microsystems GmbH, Germany), using Leica Microsystem's mouse monoclonal antibodies for ER (clone 6F11) and PR (clone 1A6). Samples were selected to cover all positivity ranges, selection based on the original diagnosis. Slides were digitalized using a *Pannoramic SCAN *digital slide scanner with Zeiss plan-apochromat objective (magnification: 20×, Numerical aperture: 0,8) and Hitachi (HV-F22CL) 3CCD progressive scan colour camera (resolution: 0,2325 μm/pixel).

### Detection calibration

The algorithm developed by the University of Pannonia, Faculty of Information Technology, Veszprém, Hungary had default detection parameters, defined based on a training sample set (i.e. 68 hematoxilin, ER, PR, Ki67, p53, p16 stained samples). The measurement of the exact deviation in the object detection and scoring quality of the system was performed in a factual environment. The system needed to be calibrated for suitable detection of ER and PR stained slides. During the calibration the detection algorithm had to set to the optical features of the digital slides created using the digital slide scanners manufactured by 3DHISTECH Ltd.

**Figure 1:**
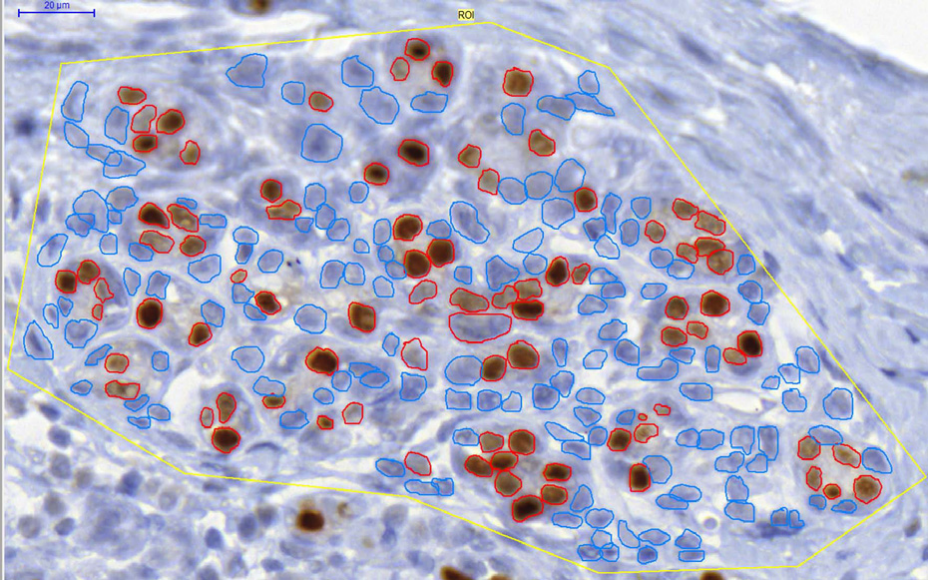
**Manual marking of cells within a ROI**. Blue = negative cells, Red = positive cells.

The calibration was performed on a selected slide set. Small areas (2-4/slide) representing each case were selected by a pathologist on the digital slides. Areas (n = 38) contained a mean number of 195 cells (min. 73- max. 489 cells). The pathologist scored each annotation on the digital slides using *PV*. The slides were later analyzed with *NQ *to compare the scores with those provided by the pathologist. The selection was followed by a manual marking (i.e. encircling using the freehand annotation option in *PV*) of each cell in the respective annotation. Different colours were used to denote the positivity classes (Figure 1). Pathologist reviewed the suitability of the marking. The process was followed by iterative analysis of each slide accompanied by the adjustment of the detection parameters of *NQ *until close reproduction of the manual encircling was obtained. The separate *.misp files, created for each slide after the most optimal detection setup, were analyzed with respect to the detection parameters (e.g. object detection, correctness of cell marking) to obtain the most appropriate setup and to create a main *.misp file that can be used on any ER or PR stained slide. After the optimal setup, all slides were re-analyzed using *NQ *and the scores were compared with those provided by the pathologist.

### Equivalence assessment

One pathologist marked from 3 to 5 biologically representative areas (regions of interest, ROI) on each digital slide, and scored the ROI on a computer monitor using *PV *only. The same ROI were later scored using *NQ *and the results of the two scorings were compared. The mean number of cells analyzed per slide was 5069 (min. 2,780-max. 19,740). The Allred scoring [18] was used by the pathologists to score the slides, and the same scoring was incorporated into *NQ*. This widely accepted and used [19,20] semi-quantitative scoring system takes into account the percent of the positively stained tumour cells and the staining intensity of the cells. The percent of positively stained tumour cells represent the Positivity Score, ranging from 0 to 5 (i.e 0 = no stained cell, 1 = 1/100 stained cells, 2 = 1/10 stained cells, 3 = 1/3 stained cells, 4 = 2/3 stained cells, 5 = up to 100% stained cells). The staining intensity, defined as the Intensity Score, ranges from 0 (i.e. no staining) to 3 (i.e. strong staining). The two scores are added to obtain the Total Score that ranges from 0 to 8, and represents positive tumours if equals or exceeds 3 [18].

### Statistics

Agreement between the pathologist's scores of digital slides and scores obtained using *NQ *was analyzed using Cohen's kappa. The strength of agreement of the kappa statistic was interpreted using the classes proposed by Landis and Koch [21] as follows: <0.00 = poor agreement, 0.00-0.20 = slight agreement, 0.21-0.40 = fair agreement, 0.41-0.60 = moderate agreement, 0.61-0.80 = substantial agreement, 0.81-1.00 = almost perfect agreement. The strength of the agreement was additionally assessed using the Spearman rank-correlation coefficient. In order to test the clinical relevancy of the agreement, quadratic weighted kappa was calculated as well, by assigning the weight 0 for the most relevant disagreement (i.e. negative Total Score = 0, 1 or 2 vs. highly positive Total Score). The data were analyzed using the SPSS 17.0 for Windows (SPSS Inc., Chicago, USA) and MedCalc for Windows v. 11.2.1.0 (MedCalc Software, Mariakerke, Belgium) software applications.

## Results

### Detection calibration

The first step of the testing was the assessment of the object detection and scoring quality of the newly developed algorithm in *NQ*, without calibration. The measurement of the deviation in the object detection and scoring of the algorithm was performed to test for the need of calibration. The analysis of the slides provided slight agreement between the two scores if tested with Cohen's kappa (κ = 0.138, SE = 0.0815, 95%CI = -0.0217-0.298), and moderate agreement using quadratic weighted kappa (κ = 0.485, SE = 0.100). Result showed that the object detection and scoring of the algorithm was not adequate, and therefore needed to be calibrated.

**Figure 2:**
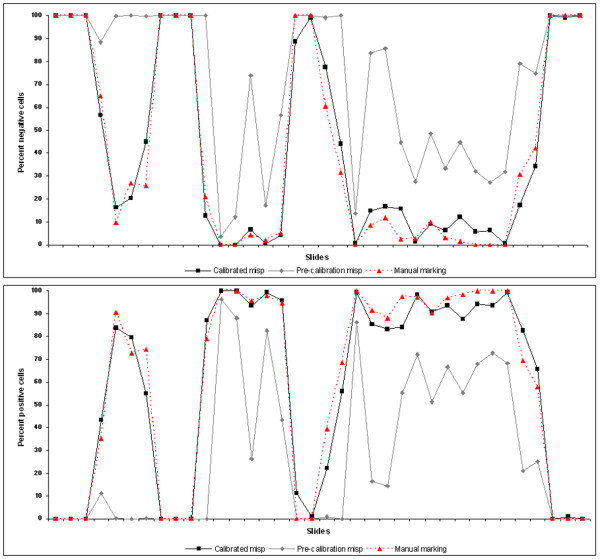
**Agreement in cell detection between the un-calibrated and calibrated algorithm and the manual encircling**.

Our approach using encircled cell nuclei (Figure 1) proved to be an adequate input to calibrate the optimal object detection and positivity determination of the algorithm (Figure 2).

**Figure 3:**
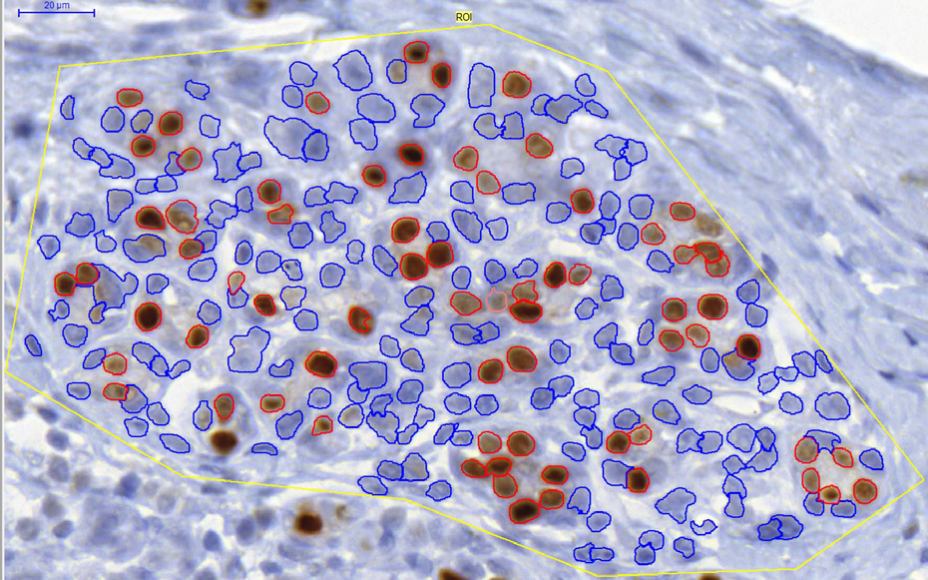
**Object detection of the calibrated *NuclearQuant *algorithm**. Blue = negative cells, Red = positive cells.

The calibrated algorithm provided an overall 87% agreement in cell detection with the manual marking, which proves a correct object detection mechanism (Figure 3). Results provided with the main *.misp file were tested against the scores provided by the pathologist. The application, similar to the pathologist, rated eight annotations as negative and 30 as positive. Almost perfect agreement between the two scoring was found with Cohen's kappa (κ = 0.859, SE = 0.0666, 95%CI = 0.729-0.990), and quadratic weighted kappa (κ = 0.986, SE = 0.162). There was a significant correlation between the pathologist's scores and the ones provided by *NQ *(Spearman's rho = 0.953, df = 37, p < 0.0001, 95% CI for rho 0.911-0.976).

### Equivalence assessment

Two slides containing 3-3 annotations each have been removed from the analysis. Both contained necrotized and ragged tissue sections, and additionally relatively intense cytoplasmic staining, thus these were not suitable for quantization using the software application.

Altogether 53 annotations have been used in the analysis. Seven annotations were scored by the pathologist as negative (i.e. Total Score between 0 and 2), 46 as positive, and the same results were obtained using *NQ*. Four discrepancies were recorded between the Positivity Scores provided by pathologist and the application. Three included down-scoring (i.e. lower *NQ *positivity score) and in one case up scoring (i.e. PS = 0 vs. 1). Similar results were obtained in the Intensity Score.

Cohen's kappa showed substantial agreement (κ = 0.795, SE = 0.0669, 95%CI = 0.664-0.926), whereas quadratic weighted kappa almost perfect agreement (κ = 0.981, SE = 0.137). There was a significant correlation between the pathologist's scores and the ones provided by *NQ *(Spearman's rho = 0.975, df = 52, p < 0.0001, 95% CI for rho 0.958-0.986).

## Discussion

Pathologist usually score IHC-stained slides with the light microscope by semi-quantitative methods, which are not standardized. Although there are several scoring guides available with colourful pictures of various slides depicting different positivity, biological samples are more heterogeneous and more 'awkward' slides exists, which are hard to be categorized. These are mainly cases with low or moderate positivity and with vesicular nuclear structure, where the chromatin is associated to the nuclear membrane and therefore the immunstaining is hard to capture. Usually a simple scoring takes several seconds, but in these problematic cases, it may take even minutes and may be un-reproducible since the result depends merely on the training and the tiredness of the pathologists' eyes and the time the pathologist deals with a single slide. By using a well calibrated and optimized automatic scoring algorithm and software, these risky cases could be scored in a standardized and reproducible way, and could additionally be documented thoroughly.

The calibration procedure applied using encircled nuclei proved an adequate input for the adjustment of the optimal object detection and the intensity thresholds to the score definition in our software application. The overall 87% cell detection agreement is regarded as excellent, considering that the slides analyzed covered the entire positivity range. This method could be used to suitably calibrate similar image analysis applications.

Following the calibration of the *NQ *algorithm the agreement with the scores provided by the pathologist was almost perfect (κ = 0.981). Similar results were reported in the literature following the assessment of different image analysis applications. Sharangpani et al. [15] found an agreement of 85% and 81% between the automatic determination of positivity/negativity of ER and PR stained cells with the subjective measurements. The agreement rate of the automated and manual scoring using higher number of samples then the ones used in our validation survey was reported to be 0.78 for ER and 0.72 for PR [10], 0.84 for ER [12] or 0.918 for ER [16]. Gokhale et al. [14] reported 95% concordance between the automated systems and observers, and Mofidi et al. [11] a highly significant correlation (r^2 ^= 0.844) between the digital scores and the manual ones.

Although the semi-automated assessment has several advantages over the manual scoring, the process has its drawbacks as well, that include i) precise and more time consuming selection of ROI to be investigated, as stromal tissue may influence the Total Score given by the application, or ii) only well prepared specimens may be quantified, as the detection algorithm may provide erroneous results if intense cytoplasmic staining is present or when the nuclei are vesicular. Still these drawbacks are superable by means of intensive training of pathologists on the use of the analysis application. The occasional misclassification errors, non-conceptional errors, arising from poorly stained samples or samples of bad quality can be solved during the review of the results by the pathologist. *NQ *provides the user with the ability to reclassify or drop individual detected objects, and thus to censor the software provided results.

By use of image analysis applications such as *NQ *presented above, the quantization of nuclear markers could be improved, made objective and reproducible. Further large clinical performance evaluation study(ies) involving several institutions and pathologists are needed to assess the safety and effectiveness of this software application with certainty before its initiation in the day-to-day pathological workflow.

## Conclusions

In conclusion, our data support the suitability of *NQ *to reliably test ER and PR status in breast cancer. The calibration procedure applied using encircled nuclei is an adequate method to calibrate image analysis applications. Further large clinical performance evaluation studies should be performed to further characterize the software application and prove its diagnostic validity.

## Competing interests

GK, DSz, VJ, PG, LF are employees of 3DHISTECH Ltd. BM is the owner of 3DHISTECH Ltd. GCs and LC were financially supported by 3DHISTECH Ltd. to develop the detection algorithm included in the *NuclearQuant *software.

## Authors' contributions

LK, VJ and GK planned the calibration. LK, TM, TK constructed the majority of the manuscript and performed the statistical analysis. TM and TK were the pathologist scoring the cases. GK, DSz, VJ, PG, LF, BM planned and developed the *NQ *software. GCs and LC develop the detection algorithm. All authors read and approved the final manuscript.
